# Impact of Hepatic Portal Venous Gas on the Prognosis of Traumatic Out-of-Hospital Cardiac Arrest: A Reason to Consider Terminating Cardiopulmonary Resuscitation

**DOI:** 10.1155/2024/7756946

**Published:** 2024-08-12

**Authors:** Seok Ran Yeom, Mun Ki Min, Dae Sup Lee, Min Jee Lee, Mo Se Chun, Sung Wook Park, Wook Tae Yang

**Affiliations:** Department of Emergency Medicine School of Medicine Pusan National University, Republic of Korea

## Abstract

**Background:**

We evaluated the prognosis of traumatic out-of-hospital cardiac arrest (OHCA) by assessing the presence of hepatic portal vein gas (HPVG) observed in ultrasound (US) or point-of-care ultrasonography (POCUS) performed during CPR. Furthermore, we aimed to understand the role of HPVG in decision-making regarding CPR discontinuation or withholding in traumatic OHCA.

**Methods:**

The retrospective study was conducted at the level 1 trauma center of urban academic medical centers in South Korea. We included adult trauma OHCA patients who underwent CPR between January 1, 2020, and June 30, 2022. Data on traumatic OHCA patients who presented to the level I trauma center during this period were extracted from the hospital's electronic medical record system. The arrest data were separately managed through the hospital's electronic medical record system for quality control, specifically the arrest registry. US images or clips of the hepatic portal vasculature (HPV) during CPR were used to assess the presence of HPVG. These images were independently reviewed by two emergency medicine physicians with several years of US examination experience who were blinded to all clinical details and outcomes. We evaluated the prognosis of traumatic OHCA by assessing the presence of HPVG using the US. In addition, we analyzed the general characteristics and assessed the impact on the ROSC in traumatic OHCA.

**Results:**

Among the 383 cardiac arrest patients, 318 traumatic OHCA patients were included. The mean age was 54.9 ± 19.4 years, and most patients were male. The initial rhythm was mainly asystole, and falls were the most frequent cause of injury. The overall ROSC rate was 18.8%, with a survival rate of 7.2% at hospital discharge. Among the 50 patients who underwent a US examination of HPV, 40 showed HPVG. The HPVG group had a significantly lower ROSC rate and survival rate at ED discharge and hospital discharge compared to the group without HPVG.

**Conclusion:**

Traumatic OHCA with HPVG presents a significantly worse prognosis. This suggests that early consideration of termination or withholding of CPR may be appropriate in such cases.

## 1. Introduction

Trauma is the leading cause of death in young adults [1, 2]. Cardiac arrest is a common presentation in the emergency department (ED); however, cardiac arrest due to trauma is rare compared to other causes. Traumatic out-of-hospital cardiac arrest (OHCA) has been reported to have a low survival rate (3%) [2, 3]. However, treating it requires a considerable amount of resources and manpower despite the low survival rate [1]. This issue has also been observed in cases of prolonged cardiopulmonary resuscitation (CPR) for OHCA [4]. Rapid discontinuation or withholding of CPR is crucial for optimizing the utility of limited medical resources and considering cost implications. This is even more important in cases of traumatic OHCA, which require significantly greater resources and manpower for treatment compared to nontraumatic OHCA. However, there is a lack of clear criteria surrounding the discontinuation or withholding of CPR in such cases [5, 6].

Point-of-care ultrasonography (POCUS) has been widely used for evaluating the causes and prognoses of cardiac arrest. There have been occasional reports of POCUS revealing the presence of hepatic portal venous gas (HPVG), which manifests as small high-echoic objects moving linearly within the hepatic vessel [7, 8].

HPVG is rarely found in serious abdominal diseases (such as infant abdominal catastrophes and mesenteric artery occlusion); however, it is associated with a high mortality rate [9]. Recently, HPVG has also been detected in various clinical settings due to advancements in imaging modalities. HPVG is sometimes discovered incidentally during CPR, and several studies have reported that HPVG during cardiac arrest is associated with a poor prognosis and lower survival rate [7, 8, 10]. However, most studies of this nature have focused on nontraumatic OHCA. Moreover, studies on HPVG in patients with traumatic OHCA are scarce.

Therefore, we evaluated the prognosis of traumatic OHCA by assessing the presence of HPVG observed using ultrasound (US) or POCUS performed during CPR. Furthermore, we aimed to understand the role of HPVG in the decision to discontinue or withhold CPR for traumatic OHCA. We also analyzed the general characteristics and factors that affect the return of spontaneous circulation (ROSC) in patients with traumatic OHCA.

## 2. Methods

### 2.1. Study Design and Participants

This retrospective study was conducted at the level 1 trauma center of the urban academic medical centers in Busan, South Korea. This institution is a 1,000-bed tertiary-level university hospital.

The study protocol was reviewed and approved by the Institutional Review Board of Busan National University Hospital (approval no. 2024-0116). The requirement for informed consent was waived by the board due to the retrospective nature of the study design.

We included adult trauma patients with OHCA who underwent CPR at the trauma center between January 1, 2020, and June 30, 2022. The inclusion criteria were as follows: (1) age ≥18 years, (2) traumatic cardiac arrest, (3) OHCA, and (4) patients for whom POCUS or US imaging was performed that offered views of the hepatic and portal veins during CPR. We excluded patients with nontraumatic cardiac arrest, in-hospital arrest, repeated arrest (except for the first traumatic OHCA), those who achieved ROSC prior to arrival at the trauma center, and patients who did not undergo POCUS.

### 2.2. Data Collection and Measurement

**Figure 1:**
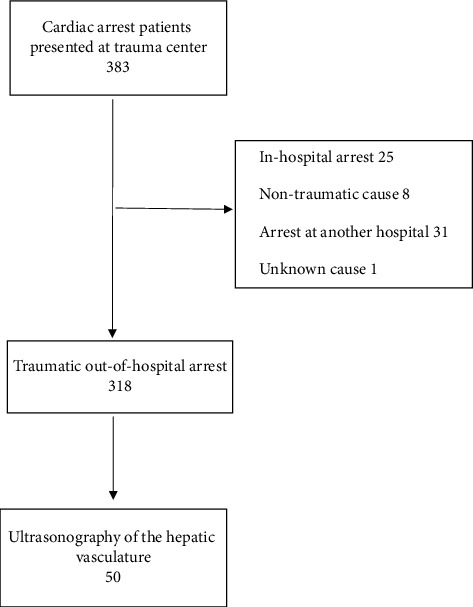
Enrollment.

Data were extracted from the hospital's electronic medical record system. The arrest data are separately managed through the arrest registry for quality control purposes. The collected medical records and arrest registries were reviewed to analyze the general characteristics of traumatic OHCA. After reviewing the extracted data, we included patients with traumatic OHCA and corresponding US findings during CPR in the ED (Figure 1).

**Figure 2:**
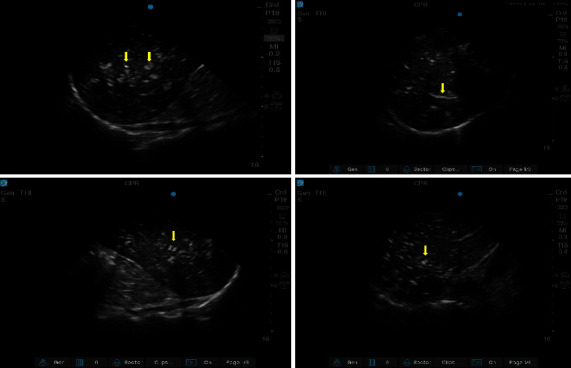
Hepatic portal vein gas observed on ultrasound in traumatic cardiac arrest. The arrow indicated hepatic portal vein gas.

The US images were independently reviewed by two emergency medicine physicians, who had performed US examinations for several years and were blinded to all of the patients' clinical details and outcomes. US images were uploaded wirelessly to a picture archiving and communication system (PACS). US images and clips were reviewed to detect HPVG. Undifferentiated hepatic gas was defined as any gas present in the hepatic portal venous system. In cases of disagreement between the physicians, the US radiologist reviewed the images. Normal liver tissue was homogenous, with medium-level echogenicity. Intravascular gas was identified as hyperechoic, sometimes mobile, foci lining the walls of the vasculature, including HPVG, which appears as small high-echoic objects moving linearly within the hepatic vessel (Figure 2) [8, 10].

During the study period, the US used in the ED was a SonoSite X-Porte system (10 MHz linear probe, 5.2 MHz convex probe, and 5.1 MHz phased array transducer; FUJIFILM SonoSite, USA).

We recorded the following demographic patient characteristics: age, sex, initial rhythm at the ED and scene, time elapsed from the scene to arrival at the hospital, bystander CPR time, CPR time of the emergency medical technician (prehospital emergency medical system), CPR time in the ED, mechanism of the traumatic accident, ROSC, result of the ED, and hospital discharge.

### 2.3. Outcomes and Statistical Analysis

We evaluated the prognoses of traumatic OHCA by assessing the presence of HPVG using the US. Furthermore, we aimed to understand the role of gas echogenicity in the hepatic portal venous system as a factor for decision-making regarding the discontinuation or withholding of CPR in cases of traumatic OHCA. Therefore, we analyzed the differences in ROSC and survival outcomes at the time of ED and hospital discharge, based on the presence of gas echogenicity within the hepatic portal vasculature. We analyzed general characteristics and assessed the impacts on ROSC in patients with traumatic OHCA.

Data were analyzed using the predictive analytics software (PASW) statistical software package for Windows, version 27 (SPSS Inc., Chicago, IL, USA), and standard descriptive summaries appropriate for the distribution of the variables were calculated. Analysis of variance (ANOVA) or Student's *t*-tests were used to compare normally distributed continuous variables among the groups. The Mann–Whitney *U* test was used for nonparametric tests. Chi-square or Fisher's exact tests were used to evaluate differences between categorical variables. Statistical significance was defined as a two-tailed *P* value of <0.05.

## 3. Results

### 3.1. General Characteristics

A total of 383 patients presented to the trauma center with cardiac arrest during the study period. Of these, 31 had received CPR at another hospital, 25 had experienced in-hospital cardiac arrest, eight had nontraumatic causes of cardiac arrest, and one had cardiac arrest due to an unknown cause and were excluded from the study. Ultimately, 318 patients with traumatic OHCA were enrolled in this study, and their general characteristics of traumatic OHCA were analyzed (Figure 1).

The mean patient age was 54.9 ± 19.4 years, with 232 (73%) males. Falls represented the most common cause of injuries, followed by motor vehicle accidents. Most initial EKGs at the hospital and at the scenes of the events showed asystole. A bystander performed CPR on 60 patients (18.8%). The mean time elapsed from the scene to arrival at the hospital was 32.8 ± 14.1 mins. Seventy patients (22%) achieved ROSC, and 49 (15.4%) survived until discharge from the ED. The survival rate at hospital discharge was 7.2% (*n* = 23; Table 1).

### 3.2. The Characteristics of ROSC in Traumatic OHCA

Seventy of the 318 patients achieved ROSC. The mean time from scene to hospital arrival was not significantly different between the ROSC and death groups. However, the CPR time at the scene or in the ED was shorter in the ROSC group. There were more cases of ventricular fibrillation (VF) or pulseless electrical activity (PEA) arrest in the ROSC group. The other variables did not differ significantly between the two groups (Table 2).

### 3.3. Difference in Prognosis Based on the Presence of Hepatic Portal Vein Gas

Among the 318 patients with traumatic OHCA, a US examination of the hepatic portal vasculature was performed in 50. Data from these 50 were analyzed to evaluate the prognosis of traumatic OHCA based on the presence of HPVG detected using the US (Figure 1).

**Figure 3:**
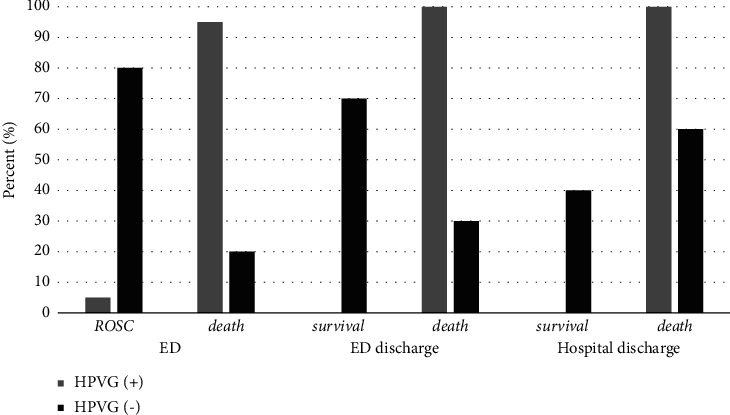
The prognostic difference based on the presence of hepatic portal vein gas in traumatic out-of-hospital arrest. ED: emergency department; ROSC: return of spontaneous circulation; HPVG: hepatic portal vein gas.

Forty patients had gas echogenicity in the hepatic portal vasculature, and 10 had normal findings. ROSC occurred in only 2 (5%) patients in the HPVG group (gas echogenicity group), whereas it occurred in 8 (80%) patients with normal liver echogenicity (normal echogenicity group). Seven patients (70%) from the normal echogenicity group survived; however, none from the gas echogenicity group survived until ED discharge. The survival rate at hospital discharge was 40% (four patients) in the normal echogenicity group (Table 3, Figure 3).

## 4. Discussion

Several studies have reported ROSC in 20–50% of cases following traumatic OHCA, with a final survival rate of approximately 3%. Among the survivors, half were in a vegetative state [11–13].

In this study, we also observed ROSC in 22% of the patients. The overall survival rate at hospital discharge was 7.2%, with severe disability or vegetative state present in 86.9%.

Traumatic OHCA has a poor prognosis; nevertheless, treatment requires significant time and numerous medical resources, which imposes a substantial burden on the healthcare system and raises concerns regarding cost-effectiveness [1, 14].

In this study, the presence of HPVG was associated with poor prognosis in cases of traumatic OHCA. The HPVG group showed an extremely low rate of ROSC in the ED, and none of the patients survived until discharge. The HPVG group had a 5% ED ROSC and a 0% discharge survival rate (normal echogenicity group: 70% ED ROSC and 40% discharge survival). The presence of HPVG in patients with traumatic OHCA was associated with a decreased risk of ED ROSC and increased overall mortality, despite ED ROSC.

HPVG was first identified in 1955 by Wolfe and Evans in six infants who were dying of abdominal catastrophes. It was thought to be a fatal sign and predictive of bowel ischemia [15–17]. More recently, HPVG has been increasingly reported in nonischemic bowel disease, with a lower mortality rate compared to ischemic bowel disease [15, 18].

HPVG has also been reported to occur during CPR in patients experiencing cardiac arrest. Lien et al. reported the presence of HPVG in 36% of patients with OHCA. Among those with HPVG, ROSC in the ED was 13%, and there were no survivors at discharge [8]. Their study included 44 patients with nontraumatic cardiac arrest. Similar to our study, it reported that the presence of HPVG was associated with a poor prognosis.

Drake et al. conducted a study on HPVG identified during POCUS in patients with nontraumatic OHCA. They reported that 54% of patients with HPVG achieved ROSC in the ED. However, only one patient survived until discharge (survival rate: 3%) [7]. Arata et al. evaluated the prognoses of 246 patients with OHCA based on the presence of HPVG, using US. Among patients with OHCA and HPVG, 15.1% achieved ROSC in the ED, and the survival rate at discharge was 1.4% [10]. All these studies reported that HPVG was associated with increased mortality.

Our study focused on patients with traumatic OHCA, distinguishing it from studies on nontraumatic OHCA. Similar to nontraumatic OHCA studies, we also observed that HPVG was associated with a poor prognosis. In our study, patients with HPVG exhibited an extremely low rate of ROCS in the ED, and none survived until discharge from the hospital. Considering that traumatic OHCA generally has a worse prognosis than nontraumatic OHCA, the presence of HPVG may indicate an even poorer outcome in traumatic OHCA.

Several factors have been proposed to predict the prognosis of cardiac arrest; however, there are currently no definitive criteria for the termination of CPR, and the decision to terminate CPR is typically made at the discretion of the attending clinical physician [5, 6]. As has been previously mentioned, traumatic OHCA requires significant medical resources; however, it is associated with a very poor prognosis. In such situations, prompt decision-making regarding early termination or withholding CPR is crucial.

Based on our study and other research findings, the early termination or withholding of CPR in traumatic OHCA when HPVG is detected may be worth considering.

However, the precise mechanism underlying the presence of portal venous gas remains unclear. Multiple theories have been proposed, including the following: (1) the influx of intraluminal gas into the portal venous system due to gastrointestinal mucosal disruption (such as bowel ischemia, infection, or bowel wall injury and others), (2) mesenteric ischemia due to low cardiac output during CPR, (3) gas-forming organisms enter the portal venous system through disrupted intestinal barriers, and (4) excess carbon dioxide production due to metabolic and respiratory acidosis during CPR [7–9, 19]. However, the exact mechanism is yet to be elucidated. Lien et al. suggested that prolonged hypoperfusion resulting from longer collapse time and advanced cardiac life support (ACLS) time causes ischemia in the intestine and severe damage to bowel mucosa. HPVG thus represents a serious condition following a common pathway rather than a specific disease [8, 20, 21].

This study had several key limitations. First, as with all retrospective reviews, there was an inherent selection bias, the potential for confounding factors, and reliance on existing medical records, which might lack some details. Second, the presence or absence of HPVG was not confirmed in all cases of traumatic OHCA. Only 50/318 (15%) cases of traumatic OHCA had confirmed HPVG. The small sample size makes it challenging to generalize these results. Lastly, within the group with confirmed HPVG, the group without HPVG (10 patients) was significantly smaller than the group with HPVG (40 patients), which may affect the robustness of the statistical comparisons.

## 5. Conclusion

Traumatic OHCA has a poor prognosis, and the presence of HPVG in traumatic OHCA is associated with an extremely unfavorable prognosis. In traumatic OHCA with HPVG, it may be prudent to consider the early termination or withholding of CPR. Further studies with larger sample sizes are required to validate these findings and establish clear criteria for CPR decisions in patients with traumatic OHCA and HPVG.

## Figures and Tables

**Table 1:** General characteristics.

Characteristics	Data (*N* = 318)
Average age (yrs)	54.9 ± 19.4
Gender (*n*)	
Male Female	232 (73%) 86 (27%)
Mechanism of injury (*n*)	
Fall down Car traffic Motorcycle traffic Stab wound Etc.	158 (50%) 85 (26.8%) 29 (9.1%) 14 (4.4%) 31 (9.7%)
Initial EKG (*N*, %) Asystole Pulseless electrical activity Ventricular fibrillation Missing	227 (71.4%) 71 (22.3%) 6 (1.9%) 14 (4.4%)
Bystander CPR (*n*, %)	60 (18.8%)
Mean elapsed time to arrival at the hospital in out-of-hospital arrest (minutes)	32.8 ± 14.1
Return of spontaneous circulation (*n*, %)	70 (22%)
Survival rate at emergency department (*n*, %)	49 (15.4%)
Survival rate at hospital discharge (*n*, %)	23 (7.2%)

**Table 2:** The characteristics of ROSC^*∗*^ in traumatic OHCA^*∗∗*^.

	ROSC	Death	*P* value
Mean elapsed time to arrival at hospital in OHCA^*∗∗*^ (minutes)	30.4 ± 13.0	33.8 ± 12.8	0.07
Bystander CPR^†^ time (mins)	7.0 ± 3.8	8.4 ± 5.7	0.46
CPR time of the emergency medical technician	19.3 ± 9.9	24.6 ± 13.4	0.001
CPR time in the emergency department	13.4 ± 9.6	20.2 ± 9.8	<0.001
Initial EKG (*N*, %) Asystole Pulseless electrical activity Ventricular fibrillation Missing	35 (15.5%) 32 (45.7%) 1 (16.6%) 2	191 (85.5%) 38 (64.3%) 5 (83.4%) 0	<0.001
Male (*n*, %)	54 (77%)	170 (72%)	0.425

^*∗*^Return of spontaneous circulation. ^*∗∗*^Out-of-hospital cardiac arrest. ^†^Cardiopulmonary resuscitation.

**Table 3:** The difference in mortality according to the presence of HPVG^*∗*^ in traumatic OHCA^*∗∗*^.

	HPVG^*∗*^ (40)	No HPVG (10)	*P* value
ROSC^†^ in ED^††^	2 (5%)	8 (80%)	<0.001
Survival at ED^††^ discharge	0 (0%)	7 (70%)	<0.001
Survival at hospital discharge	0 (0%)	4 (40%)	<0.001

^*∗*^Hepatic portal vein gas. ^*∗∗*^Out-of-hospital arrest. ^†^Return of spontaneous circulation. ^††^Emergency department.

## Data Availability

The datasets used and/or analyzed during the current study are available from the corresponding author on reasonable request.

## Additional Points

*Key Findings*. (i) ROSC: only 5% of patients in the hepatic portal venous gas (HPVG) group achieved ROSC compared to 80% in the normal echogenicity group. (ii) Survival to ED discharge: no patients with HPVG survived to ED discharge, whereas 70% with normal echogenicity did. (iii) Survival to hospital discharge: the overall survival rate at hospital discharge was 7.2%, but none of the patients with HPVG survived, compared to 40% in the normal echogenicity group. *Conclusion*. the presence of HPVG in patients with traumatic OHCA is associated with an extremely poor prognosis, with significantly lower rates of ROSC and survival compared to those without HPVG.
